# Intervening with healthcare workers’ hand hygiene compliance, knowledge, and perception in a limited-resource hospital in Indonesia: a randomized controlled trial study

**DOI:** 10.1186/s13756-017-0179-y

**Published:** 2017-02-16

**Authors:** Dewi Santosaningsih, Dewi Erikawati, Sanarto Santoso, Noorhamdani Noorhamdani, Irene Ratridewi, Didi Candradikusuma, Iin N. Chozin, Thomas E. C. J. Huwae, Gwen van der Donk, Eva van Boven, Anne F. Voor in ’t holt, Henri A. Verbrugh, Juliëtte A. Severin

**Affiliations:** 1 Department of Microbiology, Faculty of Medicine, Brawijaya University/Dr. Saiful Anwar Hospital, Malang, Indonesia; 2 Infection Prevention and Control Committee, Dr. Saiful Anwar Hospital, Malang, Indonesia; 3 Department of Medical Microbiology and Infectious Diseases, Erasmus University Medical Center, ‘s-Gravendijkwal 230, Rotterdam, 3015 CE The Netherlands

**Keywords:** Hand hygiene, Healthcare-associated infections, Indonesia

## Abstract

**Background:**

Hand hygiene is recognized as an important measure to prevent healthcare-associated infections. Hand hygiene adherence among healthcare workers is associated with their knowledge and perception. This study aimed to evaluate the effect of three different educational programs on improving hand hygiene compliance, knowledge, and perception among healthcare workers in a tertiary care hospital in Indonesia.

**Methods:**

The study was performed from May to October 2014 and divided into a pre-intervention, intervention, and post-intervention phase. This cluster randomized controlled trial allocated the implementation of three interventions to the departments, including role model training-pediatrics, active presentation-surgery, a combination of role model training and active presentation-internal medicine, and a control group-obstetrics-gynecology. Both direct observation and knowledge-perception survey of hand hygiene were performed using WHO tools.

**Results:**

Hand hygiene compliance was observed during 2,766 hand hygiene opportunities, and knowledge-perception was assessed among 196 participants in the pre-intervention and 88 in the post-intervention period. After intervention, the hand hygiene compliance rate improved significantly in pediatrics (24.1% to 43.7%; *P* < 0.001), internal medicine (5.2% to 18.5%; *P* < 0.001), and obstetrics-gynecology (10.1% to 20.5%; *P* < 0.001). The nurses’ incorrect use of hand rub while wearing gloves increased as well (*P* < 0.001). The average knowledge score improved from 5.6 (SD = 2.1) to 6.2 (SD = 1.9) (*P* < 0.05). In the perception survey, “strong smell of hand alcohol” as a reason for non-compliance increased significantly in the departments with intervention (10.1% to 22.9%; *P* = 0.021).

**Conclusion:**

The educational programs improved the hand hygiene compliance and knowledge among healthcare workers in two out of three intervention departments in a limited-resource hospital in Indonesia. Role model training had the most impact in this setting. However, adjustments to the strategy are necessary to further improve hand hygiene.

## Background

Healthcare-associated infections (HAIs) are known to be a threat in healthcare facilities, affecting morbidity, mortality, and length of stay of patients, and increase costs worldwide [1–5]. One of the most important measures to control the transmission of pathogens that may cause HAIs is hand hygiene [6, 7]. In 2005, the World Health Organization (WHO) launched the Clean Care is Safer Care campaign to encourage Member States to advocate hand hygiene. To support local improvement, a range of tools was published that were based on a multi-modal strategy with the following five components: system change, training and education, evaluation and feedback, reminders in the workplace, and institutional safety climate [1, 8]. Although the WHO guidelines and tools were designed in a way that would be of use in any setting regardless of the resources available and the cultural background, it was recognized that adaptation according to local needs, resources, and settings would be necessary [6]. Especially in developing countries, hand hygiene improvement requires a different approach than in developed countries [9]. In Indonesia, a low-middle income country, many efforts have been made over the past decade to improve the overall quality of healthcare including a national hospital accreditation program that incorporates infection control components. However, hospitals are still facing problems that typically occur in a developing country, such as overcrowding of wards and shortage of certain supplies [10, 11]. It is unknown which of the elements of the WHO multi-modal approach would have the greatest impact on the improvement of hand hygiene in such a setting [8]. Additionally, there is also only limited data on hand hygiene barriers [12]. This kind of information is necessary to redesign the approach into a suitable and feasible program for Indonesia and similar countries. This study aimed to assess the healthcare workers’ (HCWs’) hand hygiene compliance, knowledge, and perception in a limited-resource hospital in Indonesia before and after the implementation of three different educational programs.

## Methods

### Setting

The study was performed in Dr. Saiful Anwar hospital, a 902-bed tertiary care hospital, in Malang, Indonesia. In this hospital, there are four classes of care, including VIP (very important person), class I, II and III related to the room and care facilities. In this study, four departments including pediatrics, surgery, internal medicine, and obstetrics-gynecology (obst-gyn) were involved with characteristics as presented in Table 1. The alcohol-based hand rub that is used in the hospital is produced by the hospital pharmacist according to the WHO formulation II^6^. A hand rub container was attached to the footboard of each bed and next to each entrance door.

**Table 1:** Characteristics of participating wards

Dept.	Type of ward involved	Facilities	Number of patients per room	Ratio nurse: patients	Type of intervention
IM	General ward: 4 rooms	Class I^a^ (1 room)	1	1:5	Active presentations and role model training
		Class II^b^ (1 room)	7–8	1:8	
		Class III^c^ (2 rooms)	30	1:8	
SUR	Acute surgery unit: 1 room	Class III^c^	30	1:4	Active presentations
	General ward: 1 room	Class II^b^	7–8	1:6	
OBG	General ward: 2 rooms	Class III^c^	30	1:5	No intervention (control group)
PED	High care unit: 1 room	Class II^b^	14	1:2	Role model training
	Neonatology ward: 6 rooms	Class II^b^	7	1:5	

*Abbreviations*: *Dept.* Department, *IM* internal medicine, *SUR* surgery, *OBG* obstetrics-gynecology, *PED* pediatrics

^a^Patients have to share the bathroom with another patient; ^b^patients share a bathroom together; ^c^only one bathroom per room

The Dr. Saiful Anwar hospital has an infection prevention control team that consists of eight infection prevention control nurses (IPCN), each representing a specific ward (internal medicine, surgery, obst-gyn, pediatrics, intensive care unit, VIP unit, emergency unit, and operation room unit). These nurses work part time as an infection control practitioner and part time as a nurse providing patient care in the wards. The IPCN coordinate a larger team of 48 infection prevention control-linked nurses (IPCLN) who are selected among senior nurses from the different wards. IPCN and IPCLN were recruited based on Pedoman Pencegahan dan Pengendalian Infeksi di Rumah Sakit dan Fasilitas Pelayanan Kesehatan Lainnya (Guideline of Infection Prevention and Control for Hospitals and other Healthcare Services) published by Ministry of Health of the Republic of Indonesia, 2008. Before the start of the study and partly during the study period, the hospital was preparing for a national hospital accreditation. Therefore, the hand hygiene procedure, according to the existing guideline, had been introduced to HCW by the IPCN and IPCLN in collaboration with the hospital accreditation team. In addition, posters presenting the hand hygiene procedures had been posted in the workplaces. Nevertheless, observations of the hand hygiene compliance had not been conducted by the infection prevention control team until the present study started.

### Design

The design of the study was a pilot cluster randomized controlled trial, with a total duration of 24 weeks. The study was divided into three phases: pre-intervention (May to June 2014; 8 weeks), intervention (July to August 2014; 8 weeks), and post-intervention (September to October 2014; 8 weeks). The interventions consisted of three different educational programs: (1) active presentations; (2) role model training; (3) a combination of active presentations and role model training. By drawing lots, the four departments were randomly assigned to either one of the three educational interventions or to no intervention (Table 1). Active presentations to the HCW were held on at least three different occasions per ward to ensure that all HCW could participate and focused on the threat of HAIs and hand hygiene procedures [6]. In the intervention with role model training, IPCLN, as role models, received training about the hand hygiene educational program focusing on hand hygiene training techniques, including active presentations, discussions, practicing the hand hygiene procedure and the observation method. The theoretical part of HAIs and their prevention through high hand hygiene compliance was also presented to IPCLN. Therefore, they were able to motivate other HCW to better adhere to the hand hygiene procedures in the ward. The combination of active presentations and role model training was executed separately from the other interventions.

The main outcome and secondary outcome of the study was the hand hygiene compliance among HCW, including doctors, nurses, and students (either nursing students or medical students), and knowledge-perception regarding HAIs and hand hygiene among HCW obtained by a survey in the pre-intervention phase compared to the post-intervention phase, respectively. The direct observation method was applied to establish hand hygiene compliance rates, because this is considered the gold standard [13]. The observations were carried out several times a week during differing time slots, but not during the weekend. Every moment of observation lasted about 30 to 60 min. The outcomes of these observations were presented as percentages of compliance representing the fraction of the number of times when hand hygiene should have taken place correctly, and the number of times it had actually taken place correctly. The hand hygiene compliance observation sheet as well as knowledge and perception questionnaires were based on the WHO tools [6]. The knowledge survey consisted of three single item and three multiple item (i.e., more than one answer) questions on the following topics: transmission of microorganisms, source of HAIs, and hand hygiene indications. A correct answer was awarded with one point, with a maximum score of 12 points for 12 correct answers. A wrong answer led to one score deduction for multiple item questions, and a score of zero for a wrong answer to a single item question. The perception survey consisted of 12 yes/no questions, 13 of a 4-Likert-items scale questions (the last two points of the scale were considered as positive perception), and 3 open-ended questions, and included: intention to adhere to hand hygiene, risk of cross-transmission and HAIs related to non-compliance, social norms concerning hand hygiene, and hand hygiene methods, indications, importance, promotion, and compliance barriers. In addition, the three open-ended questions concerned the perception of HCW on the risk of patients acquiring HAIs, the hand hygiene compliance rate that should be achieved, and self-reporting of the hand hygiene compliance level.

When the HCW were providing patient care in the wards and filling out the surveys, two observers concurrently recorded their clothing regarding jewelry on the arms or fingers, long sleeves, and nail polish.

The study was approved by the medical ethics committee (No 129/EC/KEPK-JK/05/2012). Informed consent was not obtained, since it involved little risk of harm for the participants and the study was regarded as a hospital infection control program. Anonymity of HCW was guaranteed in the knowledge and perception survey.

### Statistical analysis

The overall compliance to hand hygiene with confidence intervals (95%CI) was calculated using the standard normal distribution. To assess differences in compliance and each of the WHO five moments of hand hygiene at the different departments between pre- and post-intervention, the Pearson Chi-Square statistic or the Fisher’s exact test was used when applicable. If the compliance in pre- and post-intervention at the departments with intervention (i.e., pediatrics, surgery and internal medicine) was significantly different from the department without intervention (i.e., obst-gyn), the Pearson Chi-Square statistic was used followed by the Mantel-Haenszel statistic. In addition, knowledge and perception improvement were analyzed using the independent *T*-test and the Chi-Square test, respectively. Backward multiple logistic regression analysis was performed to determine factors associated with hand hygiene compliance and included department, class, room type, nurse-to-patient ratio, moment of hand hygiene, and HCWs’ professions before and after intervention. A *P* value of < 0.05 was considered statistically significant and all analyses were performed using IBM SPSS version 21 (SPSS Inc., Chicago, IL, USA).

## Results

### Compliance to hand hygiene

During the study period, 2,766 potential hand hygiene opportunities were observed at the 4 participating departments. The overall compliance to hand hygiene was 19.5% (95%CI: 18.0 to 20.9). After intervention, the hand hygiene compliance rate increased both in the departments with intervention (i.e., pediatrics, internal medicine, and surgery) and in the department without intervention (i.e., obst-gyn) from 16.1% to 27.1% and from 10.1% to 20.5%, respectively. Departments pediatrics, internal medicine and obst-gyn improved significantly when comparing pre-intervention to post-intervention (*P* < 0.001). However, the intervention did not significantly improve hand hygiene compliance in the surgery department (*P* = 0.05). When considering the different types of HCW, hand hygiene compliance of doctors and nurses improved significantly post-intervention (*P* < 0.001), but not among students (*P* = 0.840). For the 538 opportunities with good hand hygiene compliance, it was observed that HCW used hand rub at 379 (70.6%) opportunities, whereas at 159 (29.6%) opportunities they washed their hands. We did not find a significant increase in the use of hand rub in the departments with intervention (Table 2). For the 2,228 opportunities with non-compliance, we found that HCW used hand rub while wearing gloves (GA) at 74 (3.3%) opportunities, wore gloves when it was not necessary at 157 (7.0%) opportunities, and did not perform hand hygiene at all at 1,997 (89.6%) opportunities. When comparing pre-intervention and post-intervention phases, the nurses who did not perform hand hygiene at all and wore gloves when it was not necessary decreased significantly (*P* = 0.024 and *P* = 0.046, respectively), however their use of hand rub while wearing gloves increased (*P* < 0.001). Similarly, the students who wore gloves when it was not necessary decreased but the use of hand rub while wearing gloves increased significantly (*P* < 0.001) (Table 3). With regard to clothing among nurses in the pre-intervention phase, 17% of the nurses wore jewelry, 31% of the nurses had long sleeves and 33% wore both jewelry and had long sleeves. Thus, a total of 81% did not wear appropriate clothing.

**Table 2:** Compliance and knowledge of hand hygiene in pre- and post-intervention

Department^a^	Compliance rate^b^			*P* value	Proportion of HCW based on Knowledge score group			*P* value
HCW	Overall % (95%CI)	Pre-intervention (%)	Post-intervention (%)		Score	Pre-intervention (%)	Post-intervention (%)	
PED	32.4 (28.6–36.2)	80/332 (24.1)	107/245 (43.7)	<0.001	0 – 5	30/60^d^ (50.0)	5/22 (22.7)	0.043
HR		53/80 (66.2)	70/107 (65.4)	1.000	6 – 12	30/60^d^ (50.0)	17/22 (77.3)	
HW		27/80 (33.8)	37/107 (34.6)					
Doctor		21/84 (25.0)	55/117 (47.0)	0.002	0 – 5	8/17 (47.1)	0/2 (0)	0.322
					6 – 12	9/17 (52.9)	2/2 (100)	
Nurse		23/105 (21.9)	45/87 (51.7)	<0.001	0 – 5	13/23 (56.5)	5/18 (27.8)	0.063
					6 – 12	10/23 (43.5)	13/18 (72.2)	
Student		36/143 (25.2)	7/41 (17.1)	0.280^c^	0 – 5	8/17 (47.1)	0/2 (0)	0.322
					6 – 12	9/17 (52.9)	2/2 (100)	
IM	12.3 (10.0–14.6)	19/364 (5.2)	74/399 (18.5)	<0.001	0 – 5	28/56 (50.0)	11/33 (33.3)	0.184
HR		11/19 (57.9)	42/74 (56.8)	1.000	6 – 12	28/56 (50.0)	22/33 (66.7)	
HW		8/19 (42.1)	32/74 (43.2)					
Doctor		3/40 (7.5)	10/37 (27.0)	0.032	0 – 5	5/13 (38.5)	NA	NA
					6 – 12	8/13 (61.5)	NA	
Nurse		6/180 (3.3)	55/295 (18.6)	<0.001	0 – 5	11/26 (42.3)	8/24 (33.3)	0.570
					6 – 12	15/26 (57.7)	16/24 (66.7)	
Student		11/144 (7.6)	9/67 (13.4)	0.181	0 – 5	9/12 (75.0)	2/5 (40.0)	0.280
					6 – 12	3/12 (25.0)	3/5 (60.0)	
SUR	21.3 (18.3–24.3)	83/440 (18.9)	73/293 (24.9)	0.05	0 – 5	16/33^e^ (48.5)	10/18 (55.6)	0.771
HR		69/83 (83.1)	64/73 (87.7)	0.501	6 – 12	17/33^e^ (51.5)	8/18 (44.4)	
HW		14/83 (16.9)	9/73 (12.3)					
Doctor		7/57 (12.3)	8/30 (26.7)	0.091	0 – 5 6 – 12	4/8 (50.0) 4/8 (50.0)	NA NA	NA
Nurse		31/238 (13.0)	46/118 (39.0)	<0.001	0 – 5	7/12 (58.3)	6/7 (85.7)	0.333
					6 – 12	5/12 (41.7)	1/7 (14.3)	
Student		45/145 (31.0)	19/145 (13.1)	<0.001^c^	0 – 5	2/8 (25.0)	4/11 (36.4)	1.000
					6 – 12	6/8 (75.0)	7/11 (63.6)	
OBG	14.6 (12.0–17.2)	40/395 (10.1)	61/298 (20.5)	<0.001	0 – 5	20/47^f^ (42.6)	3/15^g^ (20.0)	0.138
HR		32/40 (80.0)	38/61 (62.3)	0.078	6 – 12	27/47^f^ (57.4)	12/15^g^ (80.0)	
HW		8/40 (20.0)	23/61 (37.7)					
Doctor		0/18 (0)	3/12 (25.0)	0.054	0 – 5	5/10 (50.0)	NA	NA
					6 – 12	5/10 (50.0)	NA	
Nurse		24/173 (13.9)	30/157 (19.1)	0.199	0 – 5	2/12 (16.7)	1/7 (14.3)	1.000
					6 – 12	10/12 (83.3)	6/7 (85.7)	
Student		16/204 (7.8)	28/129 (21.7)	<0.001	0 – 5	10/21 (47.6)	2/7 (28.6)	0.662
					6 – 12	11/21 (52.4)	5/7 (71.4)	

*Abbreviations*: *IM* internal medicine, *SUR* surgery, *OBG* obstetrics-gynecology, *PED* pediatrics, *HCW* healthcare workers, *HR* handrubbing, *HW* handwashing, *NA* not available

^a^Departments of Pediatrics, Surgery and Internal Medicine with intervention, Department of Obstetrics-gynecology without intervention; ^b^the percentage of correct hand hygiene actions undertaken on moments when hand hygiene was considered necessary according to the WHO “five moments”; ^c^Significantly worse instead of significantly better; ^d^3 HCWs did not mention the profession in the questionnaire; ^e^5 HCWs did not mention the profession in the questionnaire; ^f^4 HCWs did not mention the profession in the questionnaire; ^g^1 HCW did not mention the profession in the questionnaire; score range 0-5 = 0-42% correct; score range 6-12 = 50%-100% correct

**Table 3:** Behavior of HCW at moments of non-compliance (*n* = 2,228 out of *n* = 2,766 observed moments)

Behavior	Phase	Total	HCW					
			Doctors (%)	*P* value	Nurses (%)	*P* value	Students (%)	*P* value
GA	Pre	4/1308 (0.3)	0/168 (0)	NA	1/612 (0.2)	<0.001	3/528 (0.6)	<0.001
	Post	70/920 (7.6)	0/120 (0)		41/481 (8.5)		29/319 (9.1)	
Gloves^a^	Pre	114/1308 (8.7)	1/168 (0.6)	1.000	67/612 (10.9)	0.046	46/528 (8.7)	<0.001
	Post	43/920 (4.7)	0/120 (0)		35/481 (7.3)		8/319 (2.5)	
No HH	Pre	1190/1308 (90.9)	167/168 (99.4)	1.000	544/612 (88.9)	0.024	479/528 (90.7)	0.292
	Post	807/920 (87.7)	120/120 (100.0)		405/481 (84.2)		282/319 (88.4)	

*Abbreviations*: *HCW* healthcare workers, *HH* hand hygiene, *GA* gloves and alcohol (using an alcohol based hand rub while wearing gloves), *Pre* pre-intervention, *Post* post-intervention

^a^Wearing gloves when it was not necessary

Based on the five moments of hand hygiene recommended by the WHO, the highest compliance was to moment 4 (27.4%) (i.e., after touching a patient). The lowest compliance was to moment 5 (12.2%) (i.e., after touching patient surroundings). Table 4 shows compliance considering the five moments pre-intervention and post-intervention at the four participating departments.

**Table 4:** Compliance to the five different WHO moments of hand hygiene pre-intervention and post intervention

WHO moment	Total (%)	Compliance (%)		*P* value
		Pre-intervention	Post-intervention	
1: Before	86/438 (19.6)	57/267 (21.3)	29/171 (17.0)	0.259
Pediatrics		30/89 (33.7)	9/39 (23.1)	0.298
Internal medicine		6/64 (9.4)	7/87 (8.0)	0.774
Surgery		7/67 (10.4)	11/22 (50.0)	<0.001
Obstetrics-gynecology		14/47 (29.8)	2/23 (8.7)	0.069
2: Before	16/123 (13.0)	7/87 (8.0)	9/36 (25.0)	0.017
Pediatrics		4/20 (20.0)	4/8 (50.0)	0.172
Internal medicine		0/31 (0)	2/14 (14.3)	0.092
Surgery		2/20 (10.0)	2/4 (50.0)	0.115
Obstetrics-gynecology		1/16 (6.3)	1/10 (10.0)	1.000
3: After	3/12 (25.0)	2/9 (22.2)	1/3 (33.3)	1.000
Pediatrics		0/4 (0)	1/1 (100)	0.200
Internal medicine		0/1 (0)	0/0 (0)	NA
Surgery		1/3 (33.3)	0/0 (0)	NA
Obstetrics-gynecology		1/1 (100)	0/2 (0)	0.333
4: After	299/1093 (27.4)	112/544 (20.6)	187/549 (34.1)	<0.001
Pediatrics		40/133 (30.1)	79/119 (66.4)	<0.001
Internal medicine		12/96 (12.5)	38/139 (27.3)	0.006
Surgery		42/178 (23.6)	42/180 (23.3)	0.953
Obstetrics-gynecology		18/137 (13.1)	28/78 (35.9)	<0.001
5: After	134/1100 (12.2)	45/624 (7.2)	89/476 (18.7)	<0.001
Pediatrics		6/86 (7.0)	14/45 (31.1)	<0.001
Internal medicine		2/172 (1.2)	27/159 (17.0)	<0.001
Surgery		31/172 (18.0)	18/87 (20.7)	0.605
Obstetrics-gynecology		6/194 (3.1)	30/185 (16.2)	<0.001

*Abbreviations*: *WHO* World Health Organization

1 = before touching a patient; 2 = before a procedure; 3 = after a procedure or body fluid exposure risk; 4 = after touching a patient; 5 = after touching a patient’s surroundings

### Hand hygiene compliance pre-intervention compared to post-intervention

Independent of phase (i.e., pre-intervention and post-intervention), we observed a statistically significant difference between compliance at departments obst-gyn and surgery (*P* < 0.001), and between obst-gyn and pediatrics (*P* < 0.001). When adding phase as a confounding factor, the relationships remained significant (*P* = 0.001 and *P* < 0.001, respectively). Independent of phase (i.e., pre-intervention and post-intervention), we did not observe a statistically significant difference between compliance at departments obst-gyn and internal medicine (*P* = 0.207). When adding phase as confounding factor, the relationship remained non-significant (*P* = 0.069).

### Factors associated with hand hygiene compliance

Multivariate analysis showed that when comparing the pre- and post-intervention phase, the pediatric and surgery department was significantly associated with hand hygiene compliance improvement among HCW (odds ratio [OR] 4.078 and 1.963; 95%CI 1.513-10.994 and 1.178-3.270, respectively). Other factors associated with the hand hygiene compliance at the different departments were general adult room (OR 1.710; 95%CI 1.002-2.918), class III room facilities (OR 1.993; 95%CI 1.168-3.400), WHO moment of before touching a patient and after touching a patient (OR 1.442; 95%CI 1.057-1.968 and OR 2.333; 95%CI 1.850-2.943, respectively). Professional categories being either a doctor or a nurse was also associated with hand hygiene compliance improvement in the post-intervention phase (OR 1.366; 95%CI 1.012-1.843 or OR 1.279; 95%CI 1.019-1.604) (Table 5).

**Table 5:** Multivariate analysis of the factors associated with hand hygiene compliance in the pre-and post-intervention phase

Factors	Univariate		*P* value	Multivariate		*P* value
	Number of HH compliance (%)			OR	95% CI	
	PI (*n* = 223)	PoI (*n* = 315)				
Department:			<0.001			
Obst-gyn	40 (17.9)	61 (19.4)		1		
Internal medicine	20 (9.0)	74 (23.5)		-	-	NS
Pediatric	80 (35.9)	107 (34.0)		4.078	1.513–10.994	0.005
Surgery	83 (37.2)	73 (23.2)		1.963	1.178–3.270	0.010
Class of room facilities:			<0.001			
Class I	1 (0.4)	20 (6.3)		1	-	NS
Class II	108 (48.4)	175 (55.6)		-	1.168–3.400	0.011
Class III	114 (51.1)	120 (38.1)		1.993		
Room type:			<0.001			
Neonatology	50 (22.4)	72 (22.9)		-	-	NS
General ward	92 (41.3)	192 (61.0)		1.710	1.002–2.918	0.049
High/acute care unit	81 (36.3)	51 (16.2)		1		
Ratio nurse: patients:						NS
1:2	30 (13.5)	35 (11.1)	0.015			
1:4–6	174 (78.0)	226 (71.7)				
1:8	19 (8.5)	54 (17.1)				
Moment of HH:			<0.001			
Moment 1	57 (25.6)	29 (9.2)		1.442	1.057–1.968	0.021
Moment 2	7 (3.1)	9 (2.9)			-	NS
Moment 3	2 (0.9)	1 (0.3)			-	NS
Moment 4	112 (50.2)	187 (59.4)		2.333	1.850–2.943	<0.001
Moment 5	45 (20.2)	89 (28.3)		1		
HCW categories:			<0.001	-		
Doctor	31 (13.9)	76 (24.1)		1.366	1.012–1.843	0.042
Nurse	84 (37.7)	176 (55.9)		1.279	1.019–1.604	0.034
Student	108 (48.4)	63 (20.0)		1		

*Abbreviations*: *HH* hand hygiene, *PI* pre-intervention, *PoI* post-intervention, *HCW* healthcare workers

Moment 1: before touching a patient; Moment 2: before a procedure; Moment 3: after a procedure or body fluid exposure risk; Moment 4: after touching a patient; Moment 5: after touching a patient’s surroundings

### Knowledge and perception

A total of 284 HCW participated in the knowledge and perception survey regarding hand hygiene and HAIs in the pre- (total *n* = 196; internal medicine, 56; surgery, 33; obst-gyn, 47, and pediatrics, 60) and post-intervention phase (total *n* = 88; internal medicine, 33; surgery, 18; obst-gyn, 15; pediatrics, 22). Overall, the average score was 5.8 (SD = 2.1), the median score was 6 and the mode score was 7/12, whereas the minimum and maximum score were 1/12 and 11/12, respectively. After interventions, the average of knowledge score improved from 5.6 (SD = 2.1) to 6.2 (SD = 1.9) (*P* < 0.05). We classified the knowledge score to be low level (0-5) and high level (6-12) and noted a significant increase in the proportion of high level scores in the pediatrics department (*P* < 0.05) after the intervention. There was not a significant change identified in other departments (Table 2). Also, we did not find a significant increase in the proportion of high level scores among doctors, nurses, and students in the four departments. The results of the perception survey on intention to adhere to hand hygiene, risk of cross-transmission and HAIs related to non-compliance, social norms concerning hand hygiene, hand hygiene indications, methods and promotion are presented in Table 6. In the departments with intervention, positive perception was demonstrated by 69.1 to 98.7% of HCW in the pre-intervention phase and 67.1 to 98.6% of HCW in the post-intervention phase to all perception items. The survey in the department without an intervention showed that 63.8% to 100% of HCW in the pre-intervention phase and 80 to 100% of HCW in the post-intervention phase answered with positive response. There was no significant improvement of the hand hygiene perception before and after the interventions. However, “strong smell of hand-alcohol” as a reason not to perform hand hygiene increased significantly in the departments with intervention. The perception of HCW in the departments with intervention regarding the average percentage of hospitalized patients who will develop a HAI increased significantly from 49.7 to 58.6% (*P* < 0.05) in the post-intervention phase. In addition, the self-reporting of hand hygiene compliance rate decreased from 85.5% to 75.1% (*P* < 0.001). We did not find any significant difference in the perception survey in the department without intervention between pre- and post-intervention (Table 6).

**Table 6:** Perception associated with HAIs and hand hygiene adherence among HCW between departments

Perception	No. of HCW (%)					
	Departments with intervention		*P* value	Department without intervention		*P* value
	Pre (*n* = 149)	Post (*n* = 73)		Pre (*n* = 47)	Post (*n* = 15)	
Formal training on HH within 3 years^a^	107 (71.8)	51 (70.8)	0.875	30 (63.8)	13 (86.7)	0.118
Intention to adhere to HH^a^	137 (91.9)	70 (95.9)	0.396	45 (95.7)	15 (100.0)	1.000
The impact of a HAIs on a patient’s clinical outcome^b^	115 (77.2)	64 (87.7)	0.119	41 (87.2)	14 (93.3)	0.759
Effectiveness of HH in preventing HAIs^b^	140 (94.0)	65 (89.0)	0.220	41 (87.2)	12 (80.0)	0.674
Importance of HH in the ward among all patient safety issues^b^	136 (91.3)	63 (86.3)	0.480	40 (85.1)	14 (93.3)	0.676
Performing HH as WHO recommended method^b^	126 (84.6)	62 (84.9)	0.944	45 (95.7)	13 (86.7)	0.244
Importance that the head of department attach to the HH behavior^b^	121 (81.2)	61 (83.6)	0.292	41 (87.2)	13 (86.7)	1.000
Importance that the colleagues attach to the HH behavior^b^	103 (69.1)	56 (76.7)	0.147	37 (78.7)	13 (86.7)	0.713
Importance that the patients attach to the HH behavior^b^	109 (73.2)	49 (67.1)	0.557	37 (78.7)	12 (80.0)	1.000
Effort to perform HH as WHO recommended method^b^	135 (90.6)	68 (93.2)	0.768	42 (89.4)	14 (93.3)	0.824
Reasons for HCW not to perform HH on a moment that it is expected^a^:						
a) Too much time	12 (8.2)	2 (2.8)	0.151	3 (6.5)	0	1.000
b) Not enough facilities	27 (19.0)	15 (21.1)	0.718	6 (13.6)	2 (13.3)	1.000
c) Skin dry or irritated	26 (18.6)	16 (22.5)	0.584	10 (21.7)	2 (14.3)	0.713
d) Hand-alcohol is not effective for hand hygiene	26 (18.2)	12 (16.9)	1.000	6 (13.3)	1 (6.7)	0.668
e) Strong smell of hand-alcohol	14 (10.1)	16 (22.9)	0.021	7 (15.2)	1 (7.1)	0.667
f) The hand-alcohol substance is not convenient (sticky)	25 (17.9)	13 (19.4)	0.848	7 (15.9)	5 (33.3)	0.263
g) The hand becomes sweaty	27 (19.6)	9 (13.4)	0.331	7 (15.9)	0	0.178
h) Feeling dirty hand after using hand-alcohol	11 (8.1)	4 (6.0)	0.777	6 (14.0)	1 (7.1)	0.669
Hand hygiene procedure as WHO guideline^a^:						
a) Information about five moments for HH is known well	129 (86.6)	67 (91.8)	0.369	45 (95.7)	15 (100.0)	1.000
b) Information about six steps of HH is known well	146 (98.0)	68 (93.2)	0.134	46 (97.9)	15 (100.0)	1.000
c) Know when to apply HH	143 (96.0)	69 (94.5)	0.838	46 (97.0)	15 (100.0)	1.000
d) Know how to apply HH	147 (98.7)	72 (98.6)	0.221	47 (100.0)	15 (100.0)	-
e) Enough reminders in the ward	121 (81.2)	59 (80.8)	0.998	43 (91.5)	13 (86.7)	0.626
Perception	%					
	(95% CI)					
	Departments with intervention		*P*	Department without intervention		*P*
	Pre	Post		Pre	Post	
Average percentage of hospitalized patients who will develop a HAIs	49.7 (44.9–54.5)	58.6 (52.8–64.4)	0.026	57.7 (51.6–63.8)	64.0 (51.3–76.7)	0.320
Average percentage of situations HCW perform HH when required	69.3 (65.2–73.3)	68.1 (63.4–72.8)	0.736	75.3 (70.0–80.6)	76.3 (62.8–89.9)	0.860
Percentage of situations requiring HH do the HCW actually perform HH, either by handrubbing or handwashing (self-reporting)	85.5 (82.6–88.4)	75.1 (70.5–79.7)	<0.001	81.8 (76.8–86.7)	85.3 (78.8–91.8)	0.425

*Abbreviations*: *HAIs* healthcare-associated infections, *HH* hand hygiene, *HCW* healthcare workers

^a^“yes” response; ^b^high/very high response

## Discussion

We report the first cluster randomized controlled trial evaluating the effect of three different educational programs on HCWs’ hand hygiene compliance and knowledge-perception in a limited-resource hospital in Indonesia. Particularly in our hospital, educational programs on hand hygiene were not applied regularly. Therefore, the educational programs used in this study were introduced in our hospital for the first time. In the departments with an intervention of role model training (i.e., pediatrics and internal medicine), the hand hygiene compliance improved, but only pediatrics department with the sole intervention of role model training was significantly better than the control group. The hand hygiene compliance improvement co-occurred with a statistically significant improvement of the knowledge score. Therefore, we conclude that role model training has the most impact on improving hand hygiene compliance in this setting. Erasmus et al. and other studies have also pointed out the importance of role models [14–16]. However, it is possible that the factor of positive role models is even more important in societies where job seniority plays a great role, such as in Indonesia.

The improvement in the pediatrics department might also be associated with fewer activities related to hand hygiene opportunities in patient care (*n* = 577) compared to internal medicine (*n* = 763), surgery (*n* = 733), and obst-gyn (*n* = 693). Pittet et al. reported the inverse relationship of activity level in the ward with hand hygiene compliance rate [17, 18]. The low activity level might also be associated with the improvement of hand hygiene adherence in general in wards and in rooms with class III type facilities. Overall, however, the hand hygiene compliance rate was low. Compared to Pakistan, also a low-middle income country [1], overall hand hygiene compliance rate in our study was lower. On the other hand, the HCW assured that they performed hand hygiene very well based on the perception survey (85.5% and 75.1% in the pre- and post-intervention phase, respectively). Therefore, the HCW may not change behavior [12]. Additionally, only good knowledge about the hand hygiene procedure did not lead to the high hand hygiene compliance among HCW. Other factors including awareness, action control, facilitation, social influence, attitude, self-efficacy, and intention might also be associated with the adherence to hand hygiene procedure. However, further investigation is needed [2].

Although hand hygiene compliance improved after intervention, we noted higher compliance rates after a procedure or body fluid exposure risk (although for only a low number of observed opportunities) and after touching a patient than before performing patient care. The lowest adherence was at the moment after touching a patients’ surroundings. Therefore, the reason to perform hand hygiene was more to protect the HCW themselves than patients [1, 17, 19, 20]. In addition, effectiveness of hand hygiene to prevent HAIs was hampered by inappropriate clothing such as hand-accessories and long sleeves by most HCW, so transmission of pathogens was unavoidable.

Based on healthcare profession, hand hygiene adherence improved among doctors and nurses in general, although it was not significant in the surgery department. The hand hygiene performance among students did not improve significantly, and even decreased in the surgery and pediatrics departments. This might be associated with the weekly rotation of students’ traineeships in our hospital leading to missing education programs, the attitudes of mentors and role models, curriculum enforcement, beliefs, and the use of gloves [21]. In such situations, students may transmit the pathogens causing HAIs from patient to patient [22].

Our data showed that wearing gloves regardless of the recommendation for gloves during patient care (i.e., wearing gloves when writing in the patient medical record) hampered HCWs’ hand hygiene adherence. WHO observed such misuse of gloves not only in limited-resource hospitals, but also in hospitals where gloves are widely available [6]. After intervention, wearing gloves without indication decreased but shifted to handrubbing while using gloves during patient care. Then, HCW did not change gloves between patients or between contacts of different sites on the same patient. Nurses declared that glove decontamination resulted from a limited examination gloves supply in our hospital (750 pairs per room in Class III). However, WHO does not recommend glove decontamination [6] because of material damage, which can endanger the protective function of gloves. Similar problems were encountered by the WHO in Ebola-affected countries, where gloves were frequently disinfected with chlorine solutions [23].

This study has some limitations. Firstly, the preparation of national hospital accreditation was held in the same period as this study, which may have influenced the knowledge and perception on hand hygiene among HCW. In addition, the HCW were busy preparing the accreditation, so participation in the knowledge and perception survey after intervention was limited. Secondly, the HCW may have changed behavior during hand hygiene observation because of their awareness of the observer (Hawthorn effect) [24, 25]. This could also be an additional explanation for the significant improvement in hand hygiene compliance in the control department. Thirdly, the study was performed in a tertiary academic hospital that included medical students and nursing students, in the delivery of patient care. Modification of the hand hygiene educational program is suggested when it is applied in either secondary or non-academic hospitals according to the hospital resources.

## Conclusions

In summary, role model training as part of a multi-model strategy has the most impact on knowledge and perception regarding hand hygiene and HAIs among HCW, and improves the hand hygiene compliance in a limited-resource hospital in Indonesia. However, we showed that the hand hygiene compliance rate remained rather low, therefore, the multi-modal hand hygiene strategy should be re-customized considering local resources, administrative support, and education/training focused on the barriers of non-established practice [1, 6, 26, 27].

## Abbreviations

CI: Confidence interval
GA: Gloves and alcohol handrub usage
HAIs: Healthcare-associated infections
HCW: Healthcare workers
HH: Hand hygiene
HR: Handrubbing
HW: Handwashing
IM: Internal medicine
IPCLN: Infection prevention control-linked nurses
IPCN: Infection prevention control nurses
NA: Not available
OBG: Obstetrics-gynecology
OR: Odds ratio
PED: Pediatrics
PI: Pre-intervention
PoI: Post-intervention
SD: Standard deviation
SUR: Surgery
VIP: Very important person
WHO: World Health Organization
